# Soccer and Vocational Training are Ineffective Delivery Strategies to Prevent HIV and Substance Abuse by Young, South African Men: A Cluster Randomized Controlled Trial

**DOI:** 10.1007/s10461-024-04458-0

**Published:** 2024-09-11

**Authors:** Mary Jane Rotheram-Borus, Mark Tomlinson, Jackie Stewart, Zwelibanzi Skiti, Stephan Rabie, Jason Wang, Ellen Almirol, Lodewyk Vogel, Joan Christodoulou, Robert E. Weiss

**Affiliations:** 1 Department of Psychiatry & Biobehavioral Sciences, Semel Institute, University of California Los Angeles, 10920 Wilshire Blvd., Suite 350, Los Angeles, CA 90024 USA; 2 Institute for Life Course Health Research, Department of Global Health, Stellenbosch University, P O Box 241, Cape Town, 8000 South Africa; 3 School of Nursing and Midwifery, Queens University, Belfast, UK; 4 Department of Psychiatry and Mental Health, HIV Mental Health Research Unit, Neuroscience Institute, University of Cape Town, Anzio Drive, Observatory, 7925 South Africa; 5 Department of Biostatistics, Fielding School of Public Health, University of California Los Angeles, Los Angeles, CA 90095-1772 USA

**Keywords:** Prevention, Soccer, Vocational training, HIV, Substance abuse, Cluster randomized controlled trial, Young men, South Africa

## Abstract

HIV and substance abuse are common among young men, associated with a cluster of risk behaviors. Yet, most services addressing these challenges are delivered in setting underutilized by men and are often inconsistent with male identity. This cluster randomized controlled trial aimed to reduce multiple risk behaviors found among young men township areas on the outskirts of Cape Town, South Africa. Young men aged 18–29 years (N = 1193) across 27 neighborhoods were randomized by area to receive HIV-related skills training during either: (1) a 12-month soccer league (SL) intervention; (2) 6-month SL followed by 6 months of vocational training (VT) intervention (SL/VT, n = 9); or 3) a control condition (CC). Bayesian longitudinal mixture models were used to evaluate behaviors over time. Because we targeted multiple outcomes as our primary outcome, we analyzed if the number of significantly different outcomes between conditions exceeded chance for 13 measures over 18 months (with 83%, 76%, and 61% follow-up). Only if there were three significant benefits favoring the SL/VT over the SL would benefits be significant. Outcome measures included substance use, HIV-testing, protective sexual behaviors, violence, community engagement and mental health. Consistent participation in the SL was typically around 45% over time across conditions, however, only 17% of men completed SL/VT. There were no significant differences between conditions over time based on the number of study outcomes. These structural interventions were ineffective in addressing young men’s substance abuse and risk for HIV.

Clinical Trial Registration: This trial was prospectively registered on 24 November 2014 with ClinicalTrials.gov (NCT02358226).

## Introduction

HIV incidence is about 2.4% annually among men aged 18–29 years in sub-Saharan Africa [1], including South Africa [2]. This results in an HIV prevalence rate of about 17% among young men [3]. Yet, HIV is only one of a cluster of challenges young men face when reaching adulthood. Rates of alcohol and drug use are syndemic with HIV among young men [4–7]. About half (56%) of young, South African men have engaged in binge drinking at least once in the past 3 months [8]. Under the influence of substances, sexual behaviors are disinhibited, increasing HIV risk for young men [9]. Concurrently, violence is often precipitated by substance abuse, including violence in the context of gangs and with intimate partners [4, 10]. In this study, about one third had been previously arrested and often had mental health symptoms [11]. This study aims to reduce this cluster of risk behaviors found among young men 18–29 years old.

Currently, there is a mismatch between where and how preventive and treatment services are offered and young men's uptake of these services. Almost all HIV interventions are embedded in health clinics [12, 13], a setting underutilized by men [14]. Men underutilize not only primary health care and HIV testing services, but are less likely to adhere to prevent and treat regimens if living with HIV [15–17]. Even when men engage in health care, the experience is often off-putting to men. HIV interventions are typically designed as individual counseling delivered by young women [18], and the topics are focused on seeking care and sharing experiences [19, 20]. Seeking help is often perceived as inconsistent with male identity [21].

We identified two attractive intervention settings to deliver evidence-based, cognitive-behavioral interventions to address young men's risk acts: soccer leagues and vocational training centers. Sports are highly attractive recreational activities that almost all men enjoy [22, 23]. Sports also form a central aspect of South Africa’s identity [24]. In the context of playing soccer, young men have an opportunity to acquire healthy habits of daily living and to build positive community norms (e.g. showing up on time for soccer with no drugs/alcohol, supporting teammates to refrain from violence). Engaging in sports has also been associated with reduced rates of substance abuse and HIV risk [25–27].

Yet, when previous sports interventions have been evaluated, researchers have typically found potential benefits limited to increased knowledge regarding HIV prevention and more positive attitudes towards prevention [28]. In a pilot study delivering HIV interventions during soccer to young men, more than 80% of young men regularly attended practices/games over 6 months and reduced their substance abuse [21]. Even if they were not good players, the young men valued being part of the team [21]. Families reported that soccer occupied the young men daily, gave them respect in the community, and the Saturday games were an opportunity for the community to attend and value the young men [29]. With all men meeting together at soccer, a traditional cognitive-behavioral, HIV and substance abuse intervention program could be implemented during a half time break. It was a novel intervention delivery method that structurally addressed all young men in a neighborhood at the same time, with the hope of shifting the social determinants of HIV for an entire community. With community members sharing the intervention experience and building new norms, the power of a traditional cognitive-behavioral program.

The second structural intervention was to offer young men vocational training. In Cape Town, 57.4% of young people are unemployed [30] and it is estimated that COVID-19 has led to another 2 million unemployed young people [30]. Going to work reduces opportunities for being part of gang activities and using drugs and alcohol, in addition to providing income that improves the quality of life. The South African government has established the Services Sector Education and Training Authority (SSETA), a jobs training program operated by the Department of Labor [31]. Though there is an annual budget of more than R3 billion, fewer than 0.9% of people receiving SSETA’s vocational training are provided with any type of on-the-job training and 60% are unemployed at the program’s completion [32]. Men have often been excluded from traditional economic development programs such as cash transfers and microfinance [33, 34]. For example, the success of the Grameen Bank in Bangladesh focused the world’s attention on microfinance as a strategy for poverty alleviation, but men are excluded [35, 36]. The few times men have been studied, the programs have been highly successful [37, 38]. Yet, policies have not changed to include men in programs involving cash transfers [39].

To reduce risk behaviors over the long term, young men need pathways out of poverty, as well as the behavioral skills to protect themselves from HIV and substance abuse problems. We offered young men immediate access to vocational training, with free transportation and tools to sustain their work at graduation from vocational training—potentially a pathway out of poverty.

To test the efficacy of these structural, community-level interventions, a three-arm, clustered randomized controlled superiority trial was designed with 27 matched neighborhood areas. For every three matched areas, UCLA randomized one area to: (1) receive soccer for 12 months (SL); (2) receive soccer for six months and vocational training for the next six months (SL/VT); or (3) to be control communities (CC). We hypothesized that the young men in the CC would engage in more sexual and substance abuse risk behaviors, fewer HIV protective behaviors, and be employed less often than men in the two intervention conditions. It is not clear whether 12 month involvement in soccer and prosocial community contact would have better outcomes (less risk and more protective behaviors) than a combination of soccer/vocational training. We wanted to evaluate which intervention strategy was optimal for young men.

## Methods

### Setting and Participants

This intervention was implemented in Khayelitsha and Mfuleni, two peri-urban settlements situated on the outskirts of Cape Town, South Africa beginning in May, 2016 and ending in February 2020. Both settlements are impoverished areas in Cape Town, and characterized by high rates of unemployment and poverty, with approximately half of residents living in informal housing [40, 41].

To be included in the study, participants had to be 18–29 years old, have slept at least four nights per week for the previous two months in Khayelitsha or Mfuleni, speak isiXhosa or English, and not be under the influence of any substances or show signs of hallucinations or delusions at the baseline interview.

### Recruitment, Randomization, and Blinding

Neighborhoods were identified as clusters of 450–600 households in Khayelitsha and Mfuleni using aerial photographs, field workers’ charting of resources in each community and street-intercept surveys of male residents. Neighborhoods were matched by UCLA in clusters of three neighborhoods each based on housing type (shacks or formal dwellings), availability of electricity, water and sanitation, and density of shebeens (bars) and health clinics. Neighborhoods were separated by buffer areas of at least 1-km or natural barriers such as highways, railways or rivers.

Recruiters randomly selected the first household in an area and then systematically approached houses in concentric circles until approximately 45 households with eligible young men were identified. Assessment staff and fieldworkers were blinded to study condition. Prior to enrolment, written informed consent was obtained from all participants. Consent forms were available in English and isiXhosa. After the baseline assessment, UCLA randomized the neighborhoods in a 1:1:1 ratio to either SL, SL/VT or CC for each cluster of neighborhoods.

### Intervention

Attendance was charted for all intervention activities by research staff.

#### Soccer League

Participants in the SL condition played soccer over a 12-month period three times weekly. The HIV and substance abuse intervention program was delivered during a break half way through the soccer practices and matches. The soccer coaches were positive role models selected from adjacent communities and were trained in the foundational skills and theory common across cognitive-behavioral, evidence-based HIV prevention programs [42, 43]. The training included roleplaying life skills regarding the core messages, including reducing substance use and violent behaviors, increasing HIV testing, healthcare utilization, healthy daily routines, prosocial friendships, and money management. The health department's HIV testing unit randomly offered HIV testing at the soccer field. Rapid diagnostic tests (RDT) for alcohol, marijuana, and methamphetamine were also given randomly twice a month. Participants received R10 (< 1 USD) for each RDT and HIV test, and those testing positive for drugs/alcohol or showing drunken/high behaviors were dismissed for the day, with options to return.

#### Socer League/Vocational Training

Participants played soccer for 6 months and then were offered 6 months training in welding, computer repairs, or woodworking. Free transportation, coaching to problem solve attendance challenges, and tools to practice the trade were provided free-of-charge at graduation. After three clusters were offered welding, there were a series of thefts of expensive equipment which eliminated the welding option.

#### Control Communities

Young men were referred to local non-government organizations and health care clinics for services.

### Procedures

Young men from adjacent neighborhoods were trained as interviewers, certified on the assessment measures, and the quality of their work was monitored over time. Interviewers were blinded to intervention condition, because assessments were conducted at a research site unrelated to the neighborhoods. Data were collected on mobile phones running *Mobenzi*, an electronic survey software package [41].

### Study Measures

Demographics and current status include age, the highest level of education achieved, employment status, partnership status, parental household, monthly income > ZAR 500 (about $30 USD), type of housing, presence of water on the property (or not), flush toilets, and electricity on site. Food insecurity was assessed using one item, the number of days going hungry in the past week, from the Household Food Insecurity Access Scale (HFIAS). This item is highly correlated with the nine-item scale among South Africans [44]. Recent suicide attempts (last three months) were reported as present (1) or not (0).

*Lifetime Historical risks* assessed at baseline:

HIV testing ever: yes (1) or no (0). While recent testing was initially listed as an outcome, rates of recent testing were confounded by testing being offered at soccer practices and during the assessments and, therefore, were not included as an outcome measure.

Sexually transmitted infections ever: yes (1) or no (0).

Engaged in sexual assault ever: yes (1) or no (0).

Group violence/involvement ever: yes (1) or no (0).

Arrested ever: yes (1) or not (0). While initially listed as an outcome, it was not possible to verify this measure and it was not included as an outcome measure.

#### Outcome Measures

We have 13 outcomes measured as Present (1) or Absent (0), where 1 represents a negative outcome and 0 is a positive outcome.

Alcohol use (1) was assessed with a urine RDT that indicated use in the last 24 hours or the RDT was negative (0).

Problematic alcohol use was assessed as 1 if in the last 3 months (a) a participant drank six or more drinks in a single day at least once a month; and reported at least one symptom of (i) drinking in the morning at least once a month, (ii) had a friend/family report about events the young man could not remember, or (iii) had a friend/professional be concerned about their drinking.

For substance abuse measures, we also collected self-reports of substance use for the last three months and last two weeks. The timeframe for detecting different drugs varies, yet self-reports were highly correlated with the results of the Rapid Diagnostic Tests (RDT) (range r = 0.44–0.8).

*Marijuana* was assessed with an RDT which diagnosed use in the last 10 days as present (1) or not (0).

*Mandrax* RDT reflected use in the last 2–3 days (1) or not (0).

*Methamphetamine* RDT documented use in the last 1–2 days (1) or not (0).

*No Employment* was measured using self-reports of the number of jobs held in the last six months. Any employment during that time was coded (0) else coded (1).

*Low income* is self-reported monthly income below 1000 ZAR/month (about $66) (1) or above 1000 (0).

*Inconsistent condom use* on the last 10 sexual encounters was reported as a 1, otherwise 0.

*Concurrent partnerships* were scored as a 1 if reported in the last three months, otherwise 0.

*Violence towards women*. Intimate partner violence (IPV) against women was self-reported if participants had hit, pulled, dragged or used a weapon on a woman, or had forced themselves on a woman (1) or not (0) in the last six months.

*Arrests* were self-reported as occurring in the last six months (1) or not (0).

*Depressive symptoms.* Depression was evaluated by 20 items, each with a response range from 0 to 3, reflecting endorsement of symptoms on the Center for Epidemiologic Studies Depression (CES-D) scale. Participants with a CES-D score 16 or greater were considered to have a depressed mood (1) vs not (0). The scale has been found reliable (Cronbach’s alpha, α > 0.85) in previous research [45].

*Lack of community engagement* (1) was defined as a participant reporting not attending any community meetings, traditional ceremonies, policy forums, or community clean-ups; not assisting any elderly, helping at church, volunteering, or helping in neighborhood watch, or attending funerals in the last 6 months; otherwise (0).

At baseline we assessed mandrax, concurrent partnerships, arrests, and community engagement over each participant’s lifetime and the baseline assessment differs from follow-up assessments. Thus, analyses of mandrax, concurrent partnerships, arrests, and community engagement omit baseline and analyze data from 6, 12, and 18 months.

#### Statistical Modeling

For each outcome, we plotted the proportion of young men reporting that outcome at baseline and each follow-up visit by intervention. We averaged each participant’s reports over the up to 3 (mandrax, concurrent partnerships, arrests, and community engagement) or up to 4 visits for each outcome and plotted the fraction in histograms. We checked for differences in retention until 18 months, separately within intervention group and globally as a function of individual baseline variables.

We modeled each outcome using a Bayesian logistic random effects model with random intercepts for neighborhoods and participants. Covariates were one indicator for baseline, 2 indicators at the 6-month follow-up (combined SL and SL/VT, else CC) and 3 indicators (SL, SL/VT, and CC) each at 12 and 18 months. Inferences were differences of differences: intervention (either SL or SL/VT) outcome at 12 or at 18 months minus baseline compared to the same for CC or the combined SL and SL/VT at 6 months minus baseline compared to the same for CC at 6 months.

**Fig. 1:**
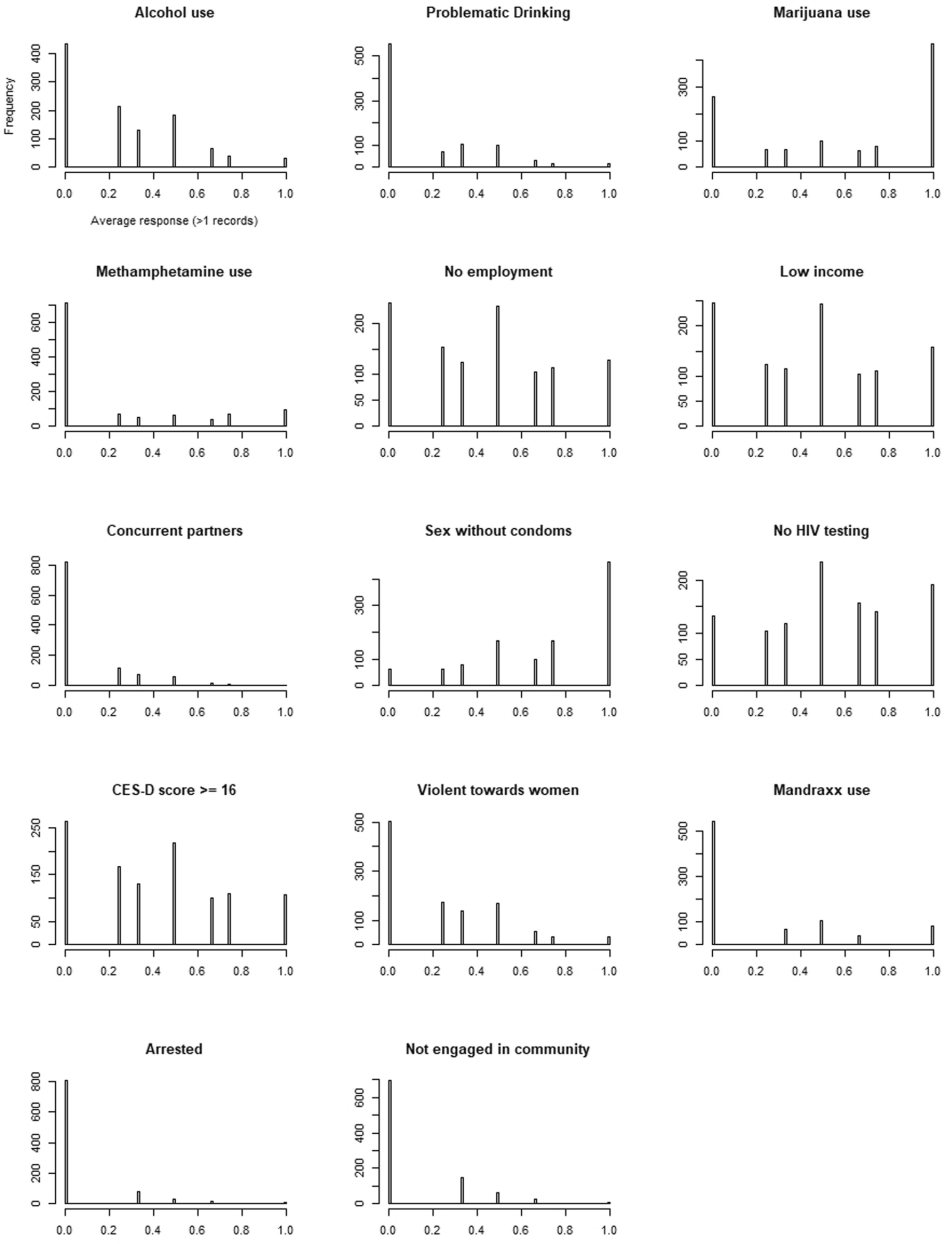
Histogram of average participant response. The number reporting each outcome as a fraction (0, .25, .33, .5, .66, .75, or 1) of their visits from 2 to 4

This model did not fit all outcomes because many participants do not ever engage in the outcome: Alcohol use, Problematic drinking, Marijuana use, Methamphetamine use, Mandrax use, Arrested, Not engaged in community, and Multiple casual partners. Figure 1 shows histograms of subject averages for all outcomes. For these 8 outcomes, we fit a mixture model; participants could either be in (a) a ‘never user’ group (did not engage in the behavior at any visit in the study), or (b) ‘ever users’ that might or might not engage in the behavior at any particular visit. Predictors of the never user group were an intercept and SL and SL/VT intervention group indicators and neighborhood random effects. For the ‘ever users’ group, we fit the same logistic random effects model as before. For outcomes fit with the mixture model, we treated inferences from the ever used group as our primary results.

We compare intervention effects at each assessment time. We report odds ratios (OR) and 95% posterior intervals (95% PI) for difference of differences at 6, 12, and 18 months from baseline for SL over CC, and at 12 and 18 months for the SL/VT intervention. For the four outcomes not assessed at baseline, we report the difference of intervention minus control at the follow-up times. We report ORs and 95% PI for SL or SL/VT for being in the never user groups compared to control.

Priors were proper but uninformative. Convergence was satisfactory. Mixture models were run in JAGS [46] in R [47] with 4 chains, burn-in of 1000 iterations, 25,000 iterations and a thin of 10 for 10,000 posterior samples. Non-mixture random-effects models were run in MCMCglmm [48] in R with 1 chain, burn-in of 10,000 iterations, then 40,000 iterations and a thin of 10 for a posterior sample of size 4000.

One SL/VT neighborhood, because of an oversight, did not receive the vocational training opportunity. We report results as intended but performed a sensitivity analysis dropping participants in this neighborhood.

We have 13 distinct though correlated outcomes, and wish to assess whether either intervention has an overall effect at any time point. Harwood and colleagues [49] proposed identifying an intervention as significantly different when it significantly affects a sufficient number of the multiple outcomes. For 13 outcomes, an intervention needs to be significantly different from SC for three or more outcomes to be declared significant overall at the alpha = 0.05 level. We applied this criterion to the SL intervention separately at 6, 12, and 18 months, and to the SL/VT intervention separately at 12 and 18 months.

*No HIV testing* was originally an outcome measure and included with the other 13 outcomes for evaluating the interventions. However, men reported testing which occurred in the context of the study; therefore, while we report on results for *No HIV testing*, we excluded this response for evaluating the intervention.

## Results

### Sample Description

**Fig. 2:**
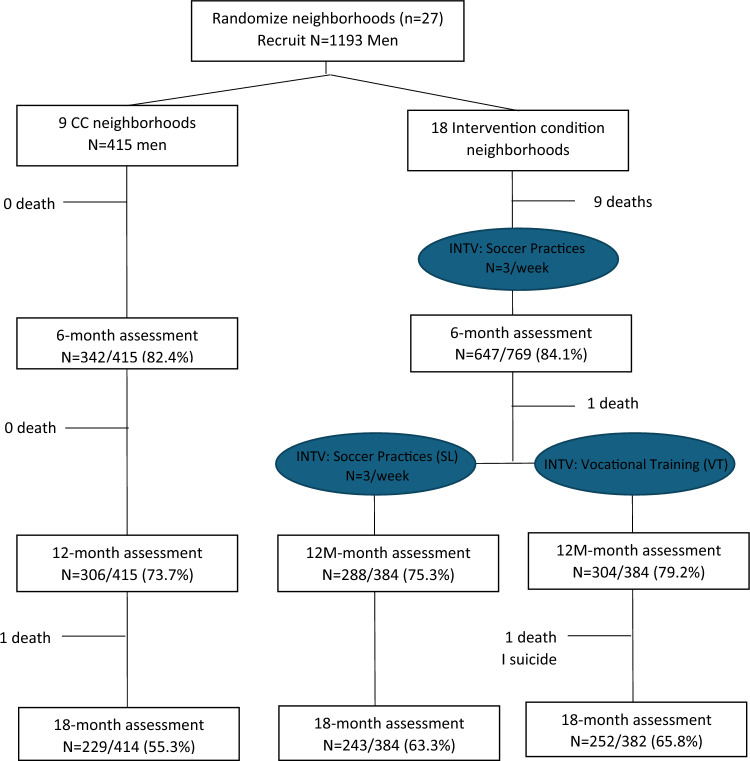
The flow of participants through the study

Between September 2016 and August 2018, we recruited and enrolled 1191 young men and conducted repeated assessments at 6 (989/1184; 83.5%), 12 (898/1183; 76%), and 18 months follow up (724/1180; 61.4%; collected during COVID lockdown) (see Fig. 2). Over the course of the study, 13 men died, shifting the sample size over time.

Table 1 summarizes sociodemographic characteristics and the outcome measures of the young men in each condition at the time of recruitment. Men were an average of 23 years old and had attended school until about 10th grade. Most lived with their parents (70%) and only about 5% were living with a partner. About 2/3 lived in formal housing, almost all had electricity, and most had access to water on the premises. Few men (3.5%) reported hunger in the past week. Sociodemographic factors were not different across conditions, as seen in Table 1.

**Table 1:** Characteristics of the sample by Soccer League (SL), Soccer plus Vocational Training (SL/VT) and Control Condition

	SL	SL/VT	Control		Total	
	(N = 389)	(N = 389)	(N = 415)		(N = 1,193)	
	n %	n %	n	%	n	%
Demographic characteristics						
Age, mean (SD)	23.0 (2.9)	22.8 (2.7)	22.9 (3.2)		22.9 (2.9)	
Highest education level, mean (SD)	10.5 (1.5)	10.4 (1.5)	10.4 (1.5)		10.4 (1.5)	
Previous Employment	284 73.0	275 70.7	284	68.4	843	70.7
In a Partnership Relationship	23 5.9	23 5.9	19	4.6	65	5.5
Living with Parents	256 65.8	261 67.1	286	68.9	803	67.3
Formal housing	190 68.8	186 67.4	206	64.4	582	64.7
Water on site	168 60.9	152 55.1	178	55.6	498	55.4
Flush toilet on site	241 79.5	218 78.9	246	76.9	705	78.4
Electricity on site	302 99.7	272 98.6	312	97.5	886	98.6
Days hungry in the past week, mean (SD)	1.6 (1.8)	1.5 (1.6)	1.5 (1.8)		1.5 (1.7)	
Recent suicide attempts	15 (8.4)	13 (6.9)	13.5 (6.5)		13.8 (7.3)	
Lifetime risks						
HIV testing	356 91.5	346 88.9	372	89.6	1074	90.0
Sexually transmitted infections	51 13.1	47 12.1	45	10.8	147	12.3
Sexual Assault*	39 10.0	39 10.0	26	6.3	104	8.7
Group Violence/Involvement	183 47.0	148 38.0	274	66.0	817	64.5
Arrests	153 39.3	133 34.2	160	38.6	446	37.4

Most of the young men (70.7%) had not been recently employed at the time of recruitment. Approximately 5% reported living with HIV. Only about 1/3 of men had a new sexual partner in the last three months, similar across conditions. About 44% reported recent violence towards women. Substance abuse was common with 31% having a positive urine RDT test for alcohol in the past 24 h. Marijuana was used by 60% and Mandrax by 23% all by RDT. Lifetime sexual assault was also significantly lower in the CC compared to the other conditions (6.3% vs 10%).

The follow-up rate at 18 months was lower than anticipated because of COVID-19 restrictions (see Appendix A). There were no significant differences between men followed up and not followed up at 18 months in any intervention group nor overall.

### Intervention Uptake

Weekly soccer attendance for the SL condition was 46.1% from months 1–6 and 42.4% from months 7–12 of the intervention. Soccer attendance was 45.3% for months 1–6 for the SL/VT condition. After 6 months of SL in the SL/VT group, 360 of 388 participants (28 had moved) were offered training in welding, computer repairs, or woodworking, In the SL/VT group, 174 (44.8%) opted for vocational training with 31 (17.8% of 174) choosing welding before the option was withdrawn, 69 (39.6%) choosing woodworking and 74 (42.5%) choosing computer training. Of those starting training, 63 (36%) graduated, with 7 (23%) graduating from plumbing, 14 (20%) graduating from woodworking and 42 (57%) graduating from computer training.

### Outcomes Over Time

**Fig. 3:**
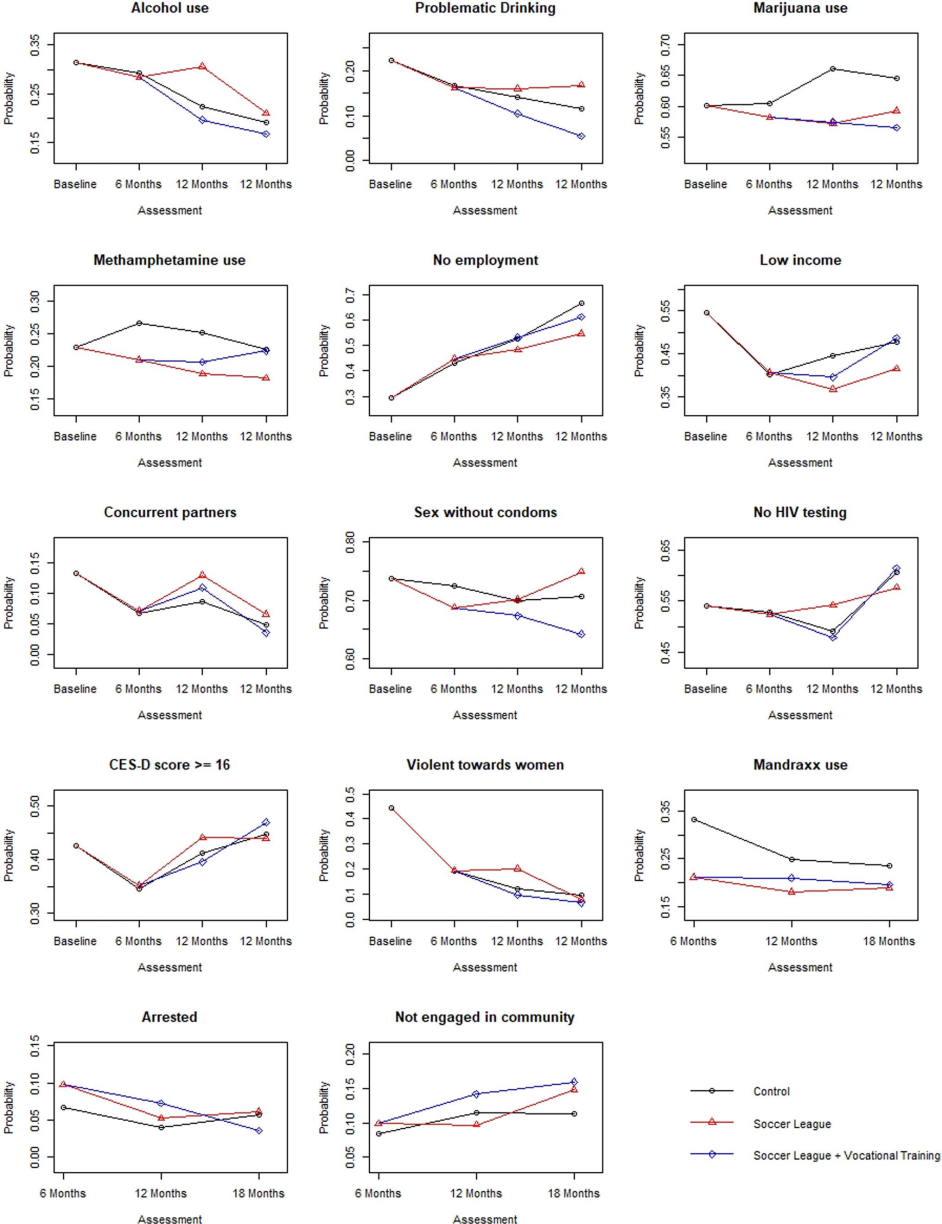
Plots of fraction of participants reporting each outcome measure in each intervention condition at baseline, and at 6, 12, and 18 months

Figure 3 plots the mean scores of each outcome by group and visit. Percentages and n’s are given in Appendix B. Table 2 summarizes comparisons of SL and SL/VT interventions to the CC for all outcomes and HIV-testing with odds ratios and 95% intervals. Outcomes analyzed with the mixture model also have columns indicating the odds ratio of being in the never user group for SL and SL/VT over CC. For SL at 6, 12, and 18 months, we found 0, 2, and 0 significantly different outcomes, while for SL/VT at 12 and 18 months we had 0 and 2 significant outcomes. Neither intervention had the required three significant outcomes to declare a significant intervention effect at any time point. The two significant 12-month SL effects were declines in marijuana (OR 0.50 95% PI (0.28, 0.91)) and Mandrax usage (OR 0.47, 95% PI (0.23, 0.97)). One SL/VT significant effect at 18 months was a decrease in problematic drinking (OR 0.42, 95% PI (0.18, 0.91)) while the other was an *increase* in methamphetamine use. For outcomes fit with the mixture model, there was one significant outcome for SL over CC with SL showing more ever alcohol users over CC (OR = 3.75, 95% PI (1.03, 15.52)). For SL/VT, the intervention group was less likely to be ever meth users as compared to CC (OR = 0.51, 95% PI (0.28, 0.85)).

**Table 2:** Odds ratios (95% CI) comparing soccer league (SL) intervention or soccer league + vocational training (SL-V) to control

Outcomes	SL 6 month	SL 12 month	SL 18 month	SL-V 12 month	SL-V 18 month	SL ( +)	SL-V ( +)
Non-mixture model							
No employment	1.34 (0.87,2.18)	1.02 (0.62,1.79)	0.62 (0.34,1.13)	1.17 (0.70,2.01)	0.76 (0.42,1.41)	NA	NA
Low income	1.51 (0.99,2.43)	1.25 (0.70,2.17)	0.87 (0.49,1.59)	1.54 (0.88,2.72)	1.65 (0.89,2.96)	NA	NA
Sex without condoms	0.93 (0.57,1.50)	1.34 (0.75,2.37)	1.85 (0.96,3.46)	0.91 (0.52,1.56)	0.74 (0.38,1.30)	NA	NA
No HIV testing	0.84 (0.56,1.28)	1.19 (0.75,1.99)	0.84 (0.50,1.50)	0.75 (0.45,1.21)	0.87 (0.50,1.53)	NA	NA
CES-D score > = 16	0.94 (0.60,1.43)	1.03 (0.61,1.66)	0.78 (0.45,1.36)	0.84 (0.50,1.38)	1.02 (0.59,1.80)	NA	NA
Violent towards women	0.90 (0.53,1.51)	1.90 (0.96,3.49)	0.59 (0.27,1.50)	0.81 (0.39,1.69)	0.66 (0.27,1.60)	NA	NA
Mixture model							
Alcohol use	0.78(0.54,1.15)	1.12(0.72,1.73)	0.77(0.46,1.29)	0.85(0.53,1.35)	0.83(0.49,1.38)	3.75(1.03,15.52)**	1.85(0.49,9.01)
Problematic drinking	0.82(0.50,1.35)	0.94(0.52,1.73)	1.34(0.67,2.61)	0.60(0.30,1.15)	0.42(0.18,0.91)*	1.91(0.57,8.28)	1.18(0.34,5.39)
Marijuana use	0.94(0.58,1.54)	0.50(0.28,0.91)*	0.83(0.44,1.57)	0.67(0.37,1.18)	0.76(0.40,1.44)	0.80(0.28,3.37)	1.03(0.36,4.29)
Methamphetamine use	0.87(0.50,1.49)	0.64(0.34,1.21)	0.75(0.37,1.48)	1.08(0.57,2.04)	2.02(1.01,4.09)*	0.66(0.36,1.17)	0.51(0.28,0.85)**
Mandraxx use	0.54(0.27,1.13)	0.47(0.23,0.97)*	0.60(0.28,1.30)	0.94(0.44,2.04)	0.95(0.42,2.16)	1.63(0.43,7.84)	0.71(0.19,3.84)
Arrested	1.16(0.55,2.41)	1.00(0.40,2.44)	0.96(0.39,2.38)	1.41(0.60,3.40)	0.55(0.20,1.41)	1.99(0.54,9.14)	1.40(0.39,6.44)
Not engaged in community	0.92(0.50,1.68)	0.74(0.37,1.47)	1.25(0.62,2.50)	1.07(0.55,2.06)	1.35(0.68,2.68)	1.47(0.36,7.26)	2.08(0.53,9.74)
Multiple casual partners	0.87(0.48,1.55)	0.86(0.43,1.71)	1.36(0.67,2.73)	0.82(0.40,1.65)	0.96(0.47,1.99)	2.09(0.56,9.97)	1.46(0.41,6.59)

( +) Odds ratio for potential use/engagement in negative outcome

^*^ Significantly better at level 0.025. ** significantly different at 0.05

^*^ p ≤ 0.05; *SD* standard deviation, *IQR* interquartile range, *RDT* rapid diagnostic test

## Discussion

Regardless of intervention condition, men continued to report substantial rates of substance abuse and risk for HIV 18 months after initiating the intervention. There were no consistent significant differences in any of the domains targeted in the intervention: sexual risk acts, substance abuse, violence, community involvement, and employment. This is disappointing in a year in which South Africa has the highest rate of HIV and a 33% increase in unemployment [30]. There are, however, a series of challenges and observations provided by these negative results.

First, even structural interventions may not succeed if interventions are not ongoing, comprehensive and possibly directly address hopelessness. We offered this intervention to all men within a community, in order to maximize the potential impact of peer support and comraderie for the acquisition of new behaviors. In particular, substance abuse programs are often designed so that young people are sent to treatment facilitates and then return to their existing networks, with high rates of substance abusers. This project was designed to shift the norms and behaviors within a community.

We did observe both relatively high uptake of the soccer intervention, and uptake increased over time. On weekly random drug tests given on the soccer field, we initially observed reductions in use. Yet, there are many holiday breaks in South Africa. In particular, at Christmas there is often a break of up to six weeks. Many young persons travel home to the Eastern Cape. During these periods, whatever substance use had decreased, returned and increased. Holidays in the entire society have alcohol as part of its celebration. This study was conducted in the Western Cape of South Africa, an area where for many generations, Black families worked on the wine farm and were paid at least partially with alcohol each week. During training, one of the first supervisors provided by a local non-profit shared his own philosophy, which did not appear atypical—*I have a paying job and I am entitled to spend my income on alcohol.* When questioned, he believed a case of hard liquor a month was reasonable. There is no way to know if this young supervisor was typical of his peers. Over 18 months there are two Christmas seasons, Easter break, and a summer vacation. We previously reported that these breaks in soccer play were characterized by patterns of significant and increasing substance abuse throughout the broader society [50]. Alcohol use is ubiquitous in some communities, and alcohol consumption forms the basis of significant amounts of peer interaction. In this context, substance use may be difficult to overcome, even when the community's family is supportive [51].

Second, more than half of the young men in the neighborhoods randomized to the vocational training intervention never registered for the training and even fewer graduated. Free transportation, food, and a promise of free tools at the end of training were unconvincing suggesting that for many men they saw little way out of unemployment. There can be many reasons for this failure to take up the programs.

A program with very similar goals to this project was mounted by this team in Uganda [52]. In Uganda, it was very easy to identify vocational training programs that offered apprenticeships. We were able to quickly identify local, one-man shops who were eager to have an apprentice—car repair, mobile phone repair, painters, construction work. In addition, there were large vocational training operations that had stable funding, in which apprenticeships were common. There was the hope that, if the trainee performed well, the trainee could join the trainer in their work. There was no such system or organization in South Africa. When we found such an organization, they were bankrupt soon. Despite South African government funding, there are few apprenticeships, and large amounts of unspent SSETA funds annually. In preparing to launch this vocational training program, we collaborated with more than six organizations. Almost all had gone out of business before we could implement the study. Each of these organizations pointed to the cumbersome government paper-work required to access SETA funds, as well as challenges enrolling and keeping young people in their programs. Even in the successful vocational training programs, we found many challenges. Our qualitative data suggest the young men found classroom-based training to be highly aversive. Yet, this was the prototypical training mode. Young men’s disinterest in vocational training may reflect their realistic appraisal that the training would not lead to a job. The models for vocational training were quite different in South Africa and lifetime unemployment rates are high. We could only offer four choices for vocational training and each started in a classroom, not on a job site. This appears unattractive to young men.

Third, ongoing stressors of young men's lives may inhibit consistency of behaviors over time. Young men often reported that they went to the Eastern Cape to "hide-out" because a negative community or interpersonal challenge occurred, and they wanted to avoid police or gangs. Those who went to the Eastern Cape were more likely to be those with fewer risk behaviors. There were also 13 deaths over the course of the study (12 of 778 in interventions; 1 of 415 in the CC). This is substantial over 18 months; we expect that the rate is even higher, but we had fewer tracking resources for young men in the CC condition. While this difference was not significant, it is possible that assembling men in groups can facilitate problem behaviors, as has previously been described by Dishion and colleagues [53].

These data highlight the importance of identifying a new approach to creating a pathway to health and employment for young men. Over time, multiple projects have failed to show soccer impacting the behaviors of either women [54, 55] or men [28, 55–60]. Sport has been used effectively to encourage HIV testing on a single event of play [61], but sport only increased knowledge and improved attitudes towards prevention when attempting to increase male circumcision [62]. Either recreational activities are not a viable intervention delivery format or projects have failed to implement effective procedures. Yet, donor agencies have significantly invested in this approach. Donors may need to routinely monitor outcomes to warrant the investments in efficacious interventions.

In contrast to the results with soccer, vocational training has been demonstrated to be successful in other LMIC [63], including LMIC in Africa [63, 64]. Yet, there are substantial data indicating that young, South African men do not reap such benefits. Substantially different vocational training approaches are likely needed.

## Conclusions

We hypothesize that both soccer and vocational training must be initiated far earlier, before the start of high school. It may be too late in early adulthood to entice men to return to school. Many had dropped out of traditional school by 10th grade. It had been a long time since their last class. Making job skills an integral part of high school and making high school a place where recreational activities such as soccer routinely occur may be a far better option than trying to create such activities in adulthood or expecting them to change behaviors. In addition, a national commitment to creating apprenticeship vocational training programs is likely a far more attractive intervention model than those currently being offered.

## Data Availability

The datasets generated and analyzed in the current study are not publicly available but will be made available from the corresponding author on reasonable request. This is because the neighborhood maps are available in other publications. If we post the data set, it might be possible to identify participants. The technology center at the Center for Community Health, headed by J. Hossell (JHossell@mednet.ucla.edu) is in charge of all distribution of data. He will be able to screen all requests, spin the data set by neighborhood identification, and mask identities. He will ensure that the data set is available beyond the employment of a specific set of investigators.

## Appendix

See Tables

**Table 3:** Comparisons between sample at Baseline and retained at 18 months by Soccer League (SL), Soccer plus Vocational Training (SL/VT) and Control Condition

	SL Retained N = 243 n%		SL Not retainer N = 146 n%		SL/VT Retained N = 253 n%		SL/VT Not retained N = 136 n%		Control Retained N = 229 n%		Control Not Retained N = 186 n%		Total Retained N = 725 n%		Total Not retained N = 468 n%					
	n	%	n	%	n	%	n	%	n	%	n	%	n	%	n		%			
Demographic characteristics																				
Age, mean (SD)	23.2 (2.8)		22.7 (3.1)		22.8 (2.8)		22.8 (2.6)		22.6 (3.0)		23.2 (3.3)		22.9 (2.9)		23.0 (3.0)					
Highest education level, mean (SD)	10.5 (1.5)		10.5 (1.4)		10.5 (1.6)		10.4 (1.5)		10.4 (1.4)		10.4 (1.7)		10.4 (1.5)		10.4 (1.6)					
Previous Employment	186 76.5		98 67.1		174 68.8		101 74.3		154 67.2		130 69.9		514	70.9	329					70.3
Married/Lives with Partner	15 6.2		8 5.5		14 5.5		9 6.6		5 2.2		13 7.0		34	4.7	30					6.4
Living with Parents	168 69.1		88 60.3		166 65.6		95 69.9		165 72.0		121 65.0		499	68.8	304					65.0
Formal housing	106/177 59.9		84/126 66.7		113/168 67.3		73/108 67.6		96/161 59.6		110/159 69.2		315/506	62.3	267/393					67.9
Water on site	93/177 52.5		75/126 59.5		88/168 52.4		64/108 59.3		83/161 51.6		95/159 59.7		264/506	52.2	234/393					59.5
Flush toilet on site	133/177 75.1		108/126 85.7		129/168 76.8		89/108 82.4		111/161 68.9		135/159 84.9		373/506	73.7	332/393					84.5
Electricity on site	177/177 100.0		125/126 99.2		166/168 98.8		106/108 98.1		155/161 96.3		157/159 98.7		498/506	98.4	388/393					98.7
Hungry in the past week (days), mean	1.6 (1.7)		1.7 (2.0)		1.5 (1.6)		1.4 (1.5)		1.5 (1.9)		1.5 (1.8)		1.5 (1.7)		1.5 (1.8)					
(SD)																				
Recent Suicide Attempt	20 8.2		10/116 8.6		16 6.3		10/106 9.4		16 7.0		11/126 8.7		52	7.2	31/348				8.9	
Lifetime risks																				
HIV Testing	223 91.8		133 91.1		226 89.3		120 88.2		202 88.2		170 91.4		651	89.8	423				90.4	
Sexually transmitted infections	35 14.4		16 11.0		29 11.5		18 13.2		26 9.1		18 9.7		90	12.4	52				11.1	
Sexual Assault	28 11.5		11 7.5		32 12.6		7 5.1		15 6.6		11 5.9		75	10.3	29			6.2		
Group Violence/Involvement	120 49.4		63 43.2		96 37.9		52 38.2		94 41.0		77 41.4		310	42.8	192			14.0		
Arrest	102 42.0		51 34.9		89 35.2		44 32.4		86 37.6		74 39.8		277	38.2	169			36.1		
Substance Use																				
RDT Alcohol Use	102 42.0		48 34.3		72 28.5		31 23.1		66 28.8		48 27.6		240	33.1	127/448			28.3		
RDT Marijuana use	146 60.1		84 57.5		149 58.9		78 57.4		142 62.0		105 60.3		437	60.3	267/448	59.6				
Self-report Mandrax/Quaalade Use																				
RDT Methamphetamine	56 23.0		32 21.9		45 17.8		30 22.1		58 25.3		47 25.3		159	21.9	109/448	24.3				

^**^p < .01; *p < .05

^3^,

**Table 4:** Summary of outcomes by intervention group and time for each outcome

	Control %	Soccer League %	Soccer League + Vocational Training %
Alcohol Use			
Baseline	28.4(114/402)	39.3(150/382)	26.7(103/386)
6 month	29.2(99/339)	30.5(99/325)	26.2(84/321)
12 month	22.4(68/303)	30.6(88/288)	20.8(59/284)
18 month	19.0(43/226)	21.0(51/243)	16.7(42/251)
Problematic alcohol use			
Baseline	20.5(64/312)	24.8(77/311)	21.3(64/301)
6 month	16.6(44/265)	16.0(41/257)	16.2(39/240)
12 month	14.1(30/213)	16.0(35/219)	11.2(21/188)
18 month	11.5(18/156)	16.8(30/179)	5.5(10/183)
Marijuana use			
Baseline	61.4(247/402)	60.2(230/382)	58.5(226/386)
6 month	60.5(205/339)	60.0(195/325)	56.4(181/321)
12 month	66.0(200/303)	57.3(165/288)	56.7(161/284)
18 Month	64.6(146/226)	59.3(144/243)	56.6(146/251
Methamphetamine use			
Baseline	26.1(105/402)	23.0(88/382)	19.4(75/386)
6 month	26.5(90/339)	22.2(72/325)	19.6(63/321)
12 month	25.1(76/303)	18.8(54/288)	20.4(58/284)
18 month	22.6(51/226)	18.1(44/243)	22.3(56/251)
Mandrax use			
6 month	33.3(75/225)	27.6(56/203)	14.7(30/204)
12 month	24.8(75/302)	18.1(52/288)	21.1(60/284)
18 month	23.5(53/226)	18.9(46/243)	19.5(49/251)
No employment			
Baseline	31.6(131/414)	27.0(105/389)	29.1(113/388)
6 month	43.1(147/341)	43.7(142/325)	46.1(148/321)
12 month	52.5(160/305)	48.3(139/288)	52.8(151/286)
18 month	66.7(152/228)	54.7(133/243)	61.4(154/251)
Low income			
Baseline	59.9(248/414)	52.4(204/389)	51.3(199/388)
6 month	40.2(137/341)	37.8(123/325)	43.6(140/321)
12 month	44.6(136/305)	36.8(106/288)	40.2(115/286)
18 month	47.8(109/228)	41.6(101/243)	48.6(122/251)
Inconsistent condom use			
Baseline	76.3(316/414)	69.9(272/389)	74.7(290/388)
6 month	72.4(247/341)	68.3(222/325)	69.2(222/321)
12 month	69.8(213/305)	70.1(202/288)	66.4(190/286)
18 month	70.6(161/228)	74.9(182/243)	64.1(161/251)
Concurrent partnerships			
6 month	27.6(94/341)	28.0(91/325)	25.2(81/321)
12 month	11.8(36/305)	12.2(35/288)	9.4(27/286)
18 month	12.7(29/228)	21.0(51/243)	14.7(37/251)
Violence towards women			
Baseline	44.0(182/414)	48.6(189/389)	40.7(158/388)
6 month	19.1(65/341)	20.9(68/325)	17.8(57/321)
12 month	11.8(36/305)	20.1(58/288)	9.4(27/286)
18 month	9.6(22/228)	7.8(19/243)	6.4(16/251)
Arrests			
6 month	6.7(23/341)	8.9(29/325)	10.6(34/321)
12 month	3.9(12/305)	5.2(15/288)	6.6(19/286)
18 month	5.7(13/228)	6.2(15/243)	3.6(9/251)
Depressive symptoms (CES-D score > = 16)			
Baseline	41.1(170/414)	44.5(173/389)	42.5(165/388)
6 month	34.6(118/341)	35.4(115/325)	34.9(112/321)
12 month	41.3(126/305)	44.1(127/288)	37.1(106/286)
18 month	44.7(102/228)	44.0(107/243)	47.0(118/251)
Lack of community engagement			
6 month	8.5(29/341)	9.2(30/325)	10.6(34/321)
12 month	11.5(35/305)	9.7(28/288)	14.7(42/286)
18 month	11.4(26/228)	14.9(36/242)	15.9(40/251)
No HIV testing			
Baseline	51.5(191/371)	52.8(188/356)	58.0(200/345)
6 month	52.8(180/341)	52.6(171/325)	52.0(167/321)
12 month	49.2(150/305)	54.2(156/288)	46.9(134/286)
18 month	60.5(138/228)	57.6(140/243)	61.4(154/251)

4.
